# Possible Effects of Climate Change on the Occurrence and Distribution of the Rare Moss *Buxbaumia viridis* in Serbia (SE Europe)

**DOI:** 10.3390/plants12030557

**Published:** 2023-01-26

**Authors:** Jovana P. Pantović, Djordje P. Božović, Marko S. Sabovljević

**Affiliations:** 1 Institute of Botany and Botanical Garden, Faculty of Biology, University of Belgrade, Takovska 43, 11000 Belgrade, Serbia; 2 Department of Botany, Institute of Biology and Ecology, Faculty of Science, Pavol Jozef Šafárik University in Košice, Mánesova 23, 040 01 Košice, Slovakia

**Keywords:** scarce species, bryophyte, the Balkans, modelling, SDM (species distribution modeling), range, global warming

## Abstract

The distribution range and occurrence of the rare and threatened epixylic moss *Buxbaumia viridis* have been reviewed in Serbia. Climatic conditions of its recent distribution in Serbia were involved in species distribution modeling and analyzed with the aim of obtaining a projection of unknown potential sites and future scenarios of its distribution dynamics. The results achieved suggest potential distribution range of the species will be significantly reduced. According to the climate change models, the habitat changes including the range loss of this species are predicted to be drastic, i.e., between 93% and 97% by the year 2050, and between 98% and 99.9% by the year 2070, affecting primarily lower elevations of its current range in Serbia. A major reason for the projected decline of the species is climate change combined with continued poor forest management.

## 1. Introduction

*Buxbaumia viridis* (Moug. ex Lam. & DC.) Brid. ex Moug. & Nestl. (syn. *B. indusiata* Brid.) is a unique moss species that occurs sporadically throughout the Northern Hemisphere, distributed from northern Fennoscandia and NW Russia south to the mountains of the northern Mediterranean region, Turkey, Caucasus, Georgia, Azerbaijan, and western North America [1]. In the region of southeastern Europe, it is known in all countries and areas [2,3], but it is very scattered and is a protected species [4]. It is considered a boreal-montane element, and is commonly found in boreal monodominant spruce forests, or less frequently in beech-fir forests. It grows mainly on decaying wood in hygrophilous and shady environments [5]. The species has a very narrow substrate receptivity, being attached to the deadwood. Additionally, not all deadwood is suitable as a habitat, but only a certain decaying stage can retain water, apart from the position, which has to be in shade/semi-shade condition. In a recent study in Austria, it was most frequently found on *Picea abies* (L.) Karst. logs and stumps, and then on *Fagus* and *Abies* substrates [6]. In Scotland, it was found on substrates of 12 different tree species and rarely on soil/humus or even live tree bark [7].

In general, *B. viridis* (green shield moss) is considered a signal species for well-preserved or sustainably maintained woodland habitats. Although it is a well-known species of old natural forests, it can also be found in artificial, planted, or heavily human-influenced forests [8].

Green shield moss is unique among the bryophyte species in that it is neotenous, living in a stage of protonema within the superficial parts of the substrate (rotting wood), and is only visible when it produces sporophyte arising out of dead wood. This species is dioecious, i.e., antheridia and archegonia are formed at the ends of different spore-originated protonemata, which also reduce the probability of fertilization and thus sporophyte formation.

This moss occurs in the assemblages of the alliance *Nowellion curvifoliae* Phil. 1965 [9]. Some of the most common accompanying species at known Serbian sites are mosses *Herzogiella seligeri* (Brid.) Z. Iwats., *Dicranum scoparium* Hedw., and *Hypnum cupressiforme* Hedw. and the liverworts *Blepharostoma trichophyllum* (L.) Dumort., *Lepidozia reptans* (L.) Dumort., *Lophocolea heterophylla* (Schrad.) Dumort., *Lophozia ventricosa* (Dicks.) Dumort., *Nowellia curvifolia* (Dicks.) Mitt., and *Riccardia palmata* (Hedw.) Carruth. (similar to elsewhere; e.g., [1,6]).

Although a rare and widely protected species, there are recent studies suggesting that its ecology and distribution are still poorly understood and possibly underestimated. Recently, there is relatively new evidence of the protonemal and gemmae stage growing independently without developing sporophyte [5]. According to the same authors, the sterile protonemal stage extended to the lower elevations in the broad-leaved forests, while sporophytes were observed only in coniferous forests at higher elevations. Seldom, it was spotted on the soil, but it is not clear whether soil type plays a role in such an occurrence or whether it is more likely to be saturated with dead wood debris. Regardless of its possible wider distribution than previously documented, this species is an important indicator of total bryophyte diversity richness on the deadwood [10]. Thorough examinations of the factors controlling its distribution and abundance are needed but scarce, although some recent studies suggest that desiccation incidence and frequency, as well as the deadwood amount present in sites, are the best predictors of *B. viridis* distribution [11]. The climate and habitat preferences are crucial to recommend this species management plans and conservation measures for this species.

Apart from the unique biological characteristic of *Buxbaumia viridis*, this epixylic forest moss is listed in Annex II of the EU Habitats Directive. As an important flagship species for deadwood-rich forests, it occurs in many protected areas. Until recently, it was considered Endangered (EN) at the overall European level [11]. However, lately, it has been the subject of a number of various surveys and monitoring programs, that have resulted in many new records and increased its distribution knowledge, for example in Czechia [8,12], Poland [13], or Slovenia [14]. Due to the recent increase in the distribution data of the species, it was recently assessed as the Least Concern (LC) at the European continental level and so reported in the new European Red List (Hodgetts et al., 2019 a, b). However, it is rare and threatened in many European countries and hence included in many national and regional Red Lists and conservation programs. For example, it is considered Critically Endangered (CR) in Italy, Montenegro, and Serbia, Endangered (EN) in Finland, Greece, Hungary, Latvia, Poland, and Romania, Vulnerable (VU) in Andorra, Britain, Czech Republic, Estonia, Slovakia, and Spain, Near Threatened (NT) in Bulgaria, Germany, Norway, Slovenia and Switzerland, ‘highly endangered’ in Austria and Data Deficient (DD) in Albania [15].

**Figure 1:**
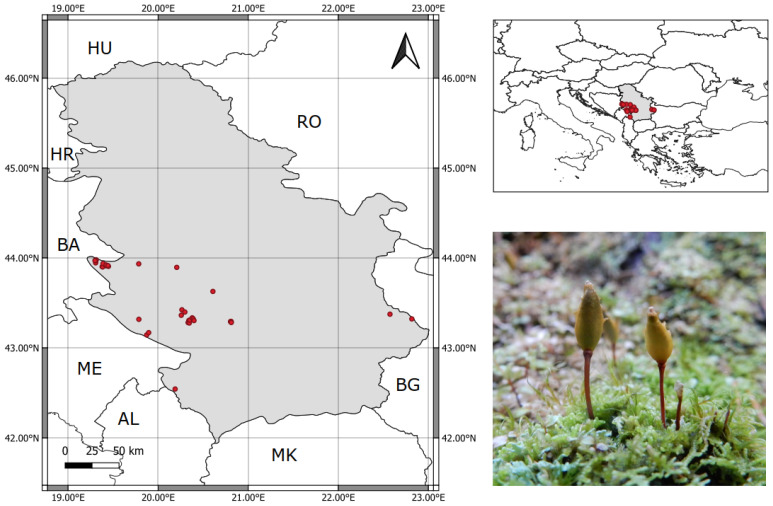
Map of Serbia (incl. Kosovo, the research area in grey) and 37 bias-corrected occurrence records used for *Buxbaumia viridis* distribution modeling (AL—Albania; BA—Bosnia & Herzegovina; BG—Bulgaria; HR—Croatia; HU—Hungary; ME—Montenegro; MK—North Macedonia; RO—Romania) (**left**). A wider view of the research area’s position in Europe (**upper right**) and the appearance of *Buxbaumia viridis* in its natural habitat (**bottom right**).

With the aim to overcome gaps in the biology and distribution of this species in Serbia (Figure 1), we applied all known biological attributes, distribution data from Serbia, and climatic conditions of the species’ recent distribution in Serbia, to (1) generate a species distribution model (SDM) and identify suitable and potential sites for this species in the bryologically under-recorded country of Serbia, from which the data about occurrence are still lacking, (2) assess the environmental variables that have the greatest impact on its distribution in the country, and finally (3) predict its range in the near future under predicted climate change models. The results should provide us with ideas for management and conservation plans as well as predict potential sites of occurrence for this species in the near future.

## 2. Results

**Figure 2:**
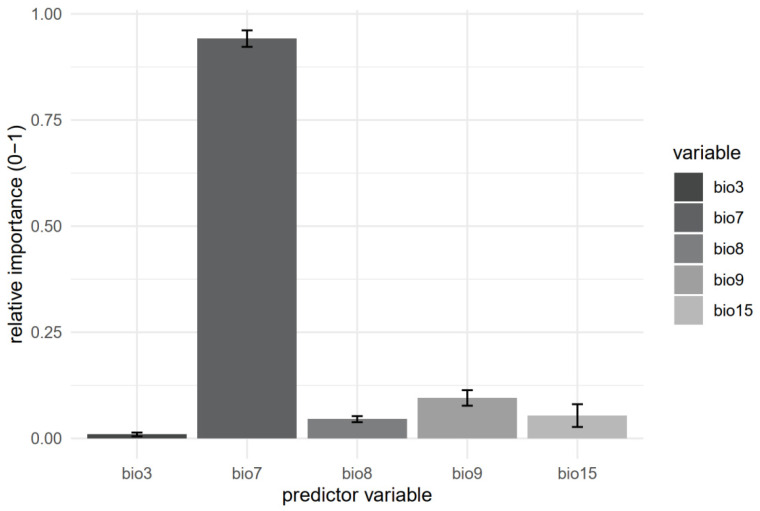
Relative contributions of five selected predictor variables to the species distribution based on ensemble modeling strategies for *Buxbaumia viridis.* The bars indicate the mean value of built ensemble models with standard errors (Bio3—Isothermality; Bio7—Temperature Annual Range; Bio8—Mean Temperature of Wettest Quarter; Bio9—Mean Temperature of Driest Quarter and Bio15—Precipitation Seasonality).

The modeling performance of the selected ensemble modeling strategy (median of probabilities) is 0.996 and 0.983 (ROC and TSS, respectively), which is considered highly significant [16], and it is the highest among the other strategies used (mean of probabilities, committee averaging and weighted sum of probabilities). This strategy also shows the highest sensitivity and specificity. Out of the final five predictive variables, the highest influence on species distribution has the temperature annual range (Bio 7) with a mean relative importance of 0.94, followed by the mean temperature of the driest quarter (Bio 9) with 0.095, precipitation seasonality (Bio 15) with 0.054, mean temperature of the wettest quarter (Bio 8) with 0.045, and isothermality (Bio 3) with 0.009 (Figure 2).

**Figure 3:**
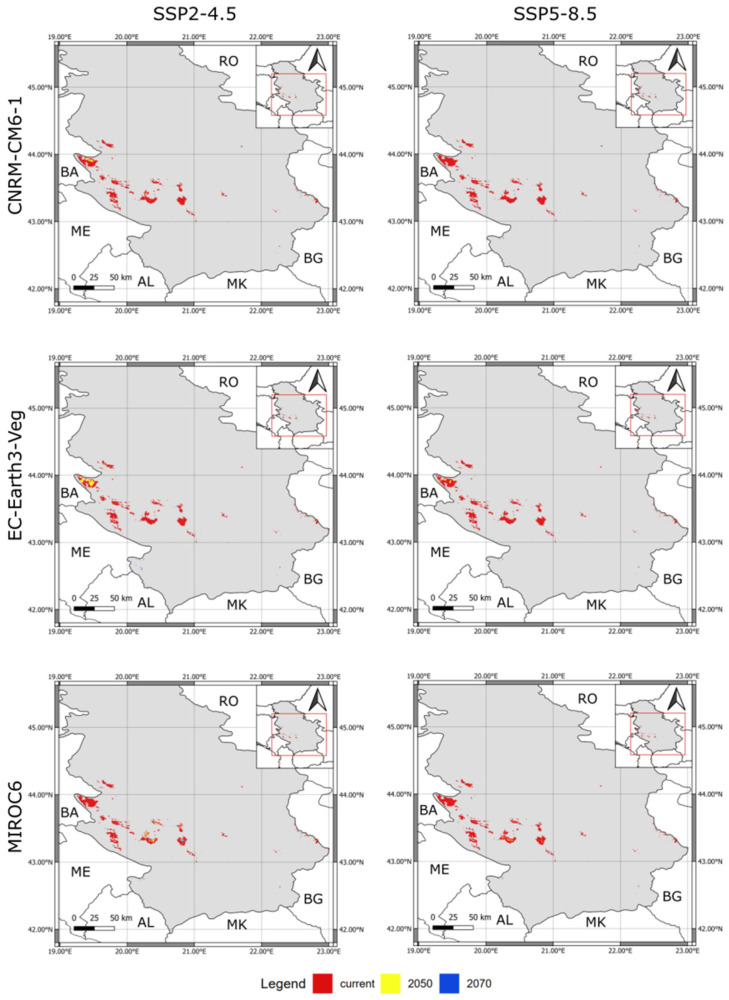
Reduction of predicted suitable range, under each GCM and SSP scenarios, for two reference periods (the 2050s and 2070s). The red color represents a current potential distribution of the species, while the colors yellow and blue represent the species’ predicted range in the years 2050 and 2070, respectively. Map insets show the relative position of the suitable area in Serbia. Country’s abbreviations (RO—Romania; BG—Bulgaria; MK—North Macedonia; AL—Albania; ME—Montenegro; BA—Bosnia & Herzegovina).

The currently suitable area for the species covers an area of approximately 1947 km^2^. Future predictions of suitable habitats for the species are shown in Figure 3. It can be seen that all future climate scenarios predict a significantly large reduction in *B. viridis* national distribution range, that is the average of ≈102.3 km^2^ of suitable surface area across the climatic models by the year 2050, and only ≈19 km^2^ by the year 2070, for SSP 2-4.5 scenario. Regarding the SSP 5.8-5 average of the predicted range across climatic models is ≈22.6 km^2^ for the year 2050 and none of the suitable territory for the species by the year 2070. It is important to emphasize that there was no difference in the percentage of habitat loss under both assumptions of unlimited and any dispersal.

**Figure 4:**
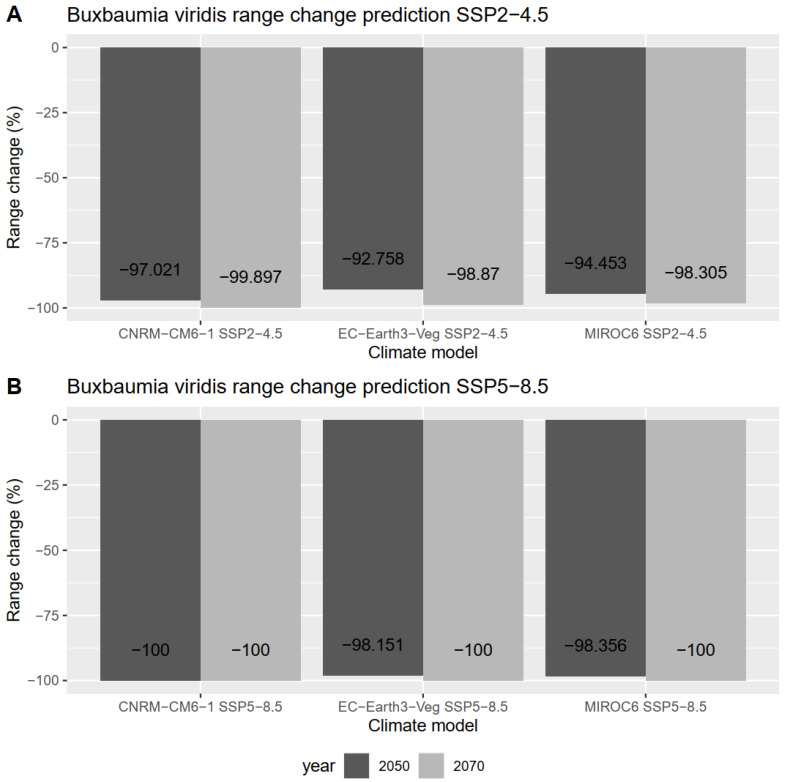
Percentage of projected range change/loss of *Buxbaumia viridis* for two reference periods (2050s and 2070s) under different GCMs and SSP scenarios (A—SSP 2-4.5 and B—SSP 5-8.5). Bars indicate values for the most accurate ensemble modeling strategy (median of probabilities).

Predictions under SSP 2.4-5 (intermediate GHG emissions) did not show a total loss of suitable habitat in any of the performed climate models (Figure 4A). The percentage of lost habitat for this emission scenario varies between ≈92.8 and 97% for the year 2050 and between ≈98.3 and 99.9% across different climatic models.

On the other hand, predictions under SSP 5.8-5 emissions (e.g., very high GHG emissions) showed total loss of suitable habitat until the year 2070 for all climatic models (Figure 4B). Total loss of suitable habitat is also predicted for one climatic model (CNRM-CM6-1) until the year 2050, while reduction of suitable habitat varies between ≈98.1 and 98.4% for the other two models (Earth3-Veg and MIROC6, respectively).

## 3. Discussion

The use of modeling techniques has proven to be very useful for understanding current distribution, predicting current range, and species records. They are becoming indispensable tools in assessing species population trends and treat status as well as the species conservation plan and program development. The importance of these models is also reflected in their ability to identify new distribution areas for important species, predict future distribution, and ultimately reduce cost and improve conservation management of species [17]. Species distribution models are particularly important considering that some species, such as *B. viridis,* can easily be overlooked in the field due to their size and seasonality [18]. Moreover, its sporophyte production, hence its finding and recording, depends on the increasingly variable climate, especially in the crucial part of the year i.e., unpredictable yearly local climate. So far, only a few studies have dealt with factors influencing its occurrence (e.g., [6,19]) or the identification of the potential (local) distribution of this significant species in Europe (e.g., [8,12,20]). Thus, in the research by Wiklund [19]., the number of occupied deadwood patches by *B. viridis* sporophyte was reduced by 73%, and the number of formed sporophytes even by 91%, during the dry year compared to the previous couple of years with higher precipitation. Low precipitation results in the desiccation of wood, even in late decay stages, thus preventing the species’ development, possibly due to inhibited fertilization or simply drying/dying out of unprotected protonemal forms. However, very little attention was being paid to the survival and distribution of this significant species under future changing climate scenarios. This is especially important as *B. viridis* is considered an indicator and signal species of the total bryophyte richness on the deadwood [10].

As a boreal-montane species, the distribution of *B. viridis* in woodlands, as expected, is shown to be under threat by climate warming. The results obtained clearly led to the conclusion that the annual temperature range is the main variable affecting the distribution of this moss. The development of the sporophyte and thus long-term dispersal and survival of the species is limited both by the summer droughts and unavailable water in the winter due to frosts as well. It appears that this species favors the conditions of a rather stable climate, both daily and annually.

The current distribution range of green shield moss is spread over mountainous parts of the country that are covered in different forest vegetation types. The calculated current extent of occurrence (EOO) of this species in Serbia is slightly smaller than 2000 km^2^. Although it appears that *B. viridis* has a relatively large range of EOO in Serbia, within this range, the species is sporadically present only in suitable and favorable microhabitats, that is, its area of occupancy (AOO) is actually relatively small. Based on the projection conducted, the species would experience extreme range decline by the year 2050, and even extinction by the year 2070, under both future climate scenarios. The loss of range is expected to affect mainly the lower elevations of the current Serbian range, whereas the area with climatically suitable conditions is predicted to shift towards the higher elevations where possible (for example in the Kopaonik Mt. and Golija Mt.), but these highest areas cover quite small surfaces in Serbia.

Indeed, overall habitat loss in fact increases the likelihood of suitable microhabitats under climate change scenarios raising concern for species survival, at least at the national level. It is worth noting that species metapopulation in Serbia is already at the southern edge of its overall European range and the survival of this metapopulation as a result of these new findings leads to increased concern for *B. viridis* at least at the regional, i.e., national level.

This study did not consider other environmental variables that may additionally influence the distribution and occurrence of the species, especially at the microhabitat scale, but the (macro)climatic factors of the area turn out to be quite drastic. However, some of these factors, such as the geological bedrock, may be of great importance for the distribution of this species. The geological bedrock is particularly important for the availability i.e., retention of the water in the habitat, mostly in the substrate, despite the seasonality of precipitation. Thus, in parts of the country with less porous substrates (e.g., siliceous rocks), moisture is present in habitats for longer periods of time, which may affect the survival of the given species in such habitats, despite to a certain extent lower altitudes and/or smaller total rainfall.

Estimating the decline and/or extinction of a species requires not only an assessment of the change in suitable habitats, but also the ability of the species to disperse [21]. In the research of Rumpf et al. [22], 38% of the plant species studied were not able to colonize all the sites climatically suitable for them. The ability of a species to colonize a suitable habitat depends on its dispersal capacity, which for bryophytes depends mainly on the size and number of spores/diaspores produced. Despite their large dispersal capacity, all bryophytes are estimated to lag behind the rate of climate change [23]. In general, scarce and fragmented microhabitats suitable for *B. viridis* within the woodland zone are probably one of the main unfavorable factors that affect the dispersal of this species. Other factors that may limit its dispersal are the removal of deadwood and the predation of sporophyte capsules in this species, which has been detected especially on young capsules [24,25].

The rarity of *B. viridis* is largely a consequence of its ecological traits such as dioeciousness, short life cycle, sensitivity to substrate desiccation, and low competitive ability [19], but also specific biannually bi-phase ontogenesis. One of the main threats to many epixylic bryophytes, including the rare *B. viridis,* is undoubtedly silviculture practices such as the removal of fallen timber in intensely managed forest areas, as well as clear deforestation of old-growth forests [6,26]. Thus, various anthropogenic activities degrade *B. viridis* habitats and/or destroy suitable microhabitats, exerting great pressure on the populations of this species. Finally, climate change (particularly rising temperatures related to the decrease in watering) also poses a serious threat to the species, negatively affecting the survival of *B. viridis* populations and causing them to migrate to higher elevations and northward in the future. With this in mind, projections for the future distribution of *B. viridis* in Serbia are generally very pessimistic. The range changes modeled in this study are only a strong reference to the urgent active need for conservation measures for *B. viridis* moss and its current habitats, including habitat protection, development of better forest management strategies, and monitoring programs for the species.

## 4. Materials and Methods

### 4.1. Study Area and Species Occurrence Data

The territory of the Republic of Serbia, a country located in southeastern Europe in the central part of the Balkan Peninsula, was selected for the study area. The climate of Serbia is quite complex and heterogeneous, because of the complex relief and different influences coming from the Atlantic, the Mediterranean, the Pannonian, and mountainous areas. Hence, its climate ranges from dry continental in the north to a more humid variant in the western part of the country, the mild continental climate in the central part of the country, while the southern parts are rather dry submediterranean variant. Moreover, the various mountain climate types are present in different regions of the country. The amount of precipitation varies depending on prevailing climatic patterns and it ranges from ca. 500 mm per year in northern Vojvodina, ca. 1000 mm in western Serbia, to more than 1500 mm in some mountainous regions. However, significant total precipitation local differences should be taken into consideration along with the precipitate amounts unevenly distributed throughout the year or during vegetation seasoning even in nearby sites.

Occurrence records of *B. viridis* in Serbia were collected from literature sources [27,28,29,30,31,32,33,34,35,36,37,38,39,40,41,42], as well as the bryophyte collection of the Herbarium of the University of Belgrade (BEOU). Altogether, 61 occurrence sites within the country were noted. Given that most SDM (species distribution modeling) methods require spatially independent data [43,44], the spatial bias reduction of the findings was necessary. It was done manually using QGIS [45], reducing it to one record per ~1 km^2^ (e.g., one presence point per spatial resolution unit of climate data). After bias reduction, 37 records were used for the final modeling (Figure 1), which is a sample size that most modeling algorithms can cope with, without a tradeoff and with significant accuracy [46,47,48].

### 4.2. Climate Data

To characterize the current climatic conditions in which *B. viridis* is present, 19 bioclimatic variables (bio1–bio19) at the spatial resolution of 30 arc second (~1 km at the equator) were downloaded from the WorldClim dataset (http://www.worldclim.org, accessed on 8 July 2022). Given the fact that bioclimatic variables are usually strongly correlated [49], collinearity reduction was performed using variance inflation factor (VIF) with usdm R package [50] in R [51]. Vifcor function finds a pair of variables with a correlation coefficient higher than 0.7 (defined threshold) and excludes one with higher VIF until no highly correlated variable pair remains. Fourteen out of 19 input variables had collinearity issues. Based on the previous procedure, the following 5 variables were selected for further tests: Bio3—Isothermality; Bio7—Temperature Annual Range; Bio8—Mean Temperature of Wettest Quarter; Bio9—Mean Temperature of Driest Quarter and Bio15—Precipitation Seasonality.

Prediction of potential future distribution of the species was based on future climate data for two reference periods: 2050s (2041–2060) and 2070s (2061–2080) under two SSPs (Shared Socio-economic Pathways) [52], SSP 2-4.5 that implies intermediate greenhouse gases emissions (CO_2_ emissions around current levels until the year 2050, after which they are decreasing without reaching net zero by the year 2100) and SSP 5-8.5 that implies very high greenhouse gasses emissions (tripled CO_2_ emissions by 2075). Total of three different CMIP6 (Coupled Model Intercomparison Project Phase 6) [53] global climate models (GCMs): CNRM-CM6-1 [54].; EC-Earth3-Veg [55] and MIROC6 [56] that were obtained from WorldClim Version 2.1 dataset [57] in 30 arc second spatial resolution for mentioned reference periods and SSPs were used. (for additional details please refer to the documents in the Supplementary Material).

### 4.3. Species Distribution Modeling

In general, it is considered that ensemble models usually outperform individual model predictions [58,59]. In that manner, the creation of an ensemble model [60] that describes a potentially suitable habitat for *B. viridis* was done using R [51] with biomod2 package (v. 4.2-1; https://github.com/biomodhub/biomod2, accessed on 6 October 2022) [61]. This package offers a variety of different modeling approaches that were used: GLM—generalized linear model [62]; GAM—generalized additive model [63]; MARS—multivariate adaptive regression splines [64]; CTA—Classification tree analysis [65]; MDA—mixture discriminant analysis [66]; ANN—artificial neural networks [67]; RF—random forests [68]; GBM—generalized boosting model [69]; MaxEnt—maximum entropy algorithm [70]; SRE—surface range envelope [71].

Most niche models require both, presence and absence data, and therefore three datasets (to prevent sampling bias) of randomly distributed pseudo-absences (10,000 points each) were generated using the *BIOMOD_FormatingData* function, using the random sampling method. Each model has been run for each pseudo-absence dataset in four repetitions with 80% of the data used for model calibration and 20% for model evaluation, using three different evaluation metrics: ROC—Relative Operating Characteristic [72], TSS—True Skill Statistics [73] and KAPPA—Cohen’s kappa coefficient [74]. The described procedure resulted in 120 models in total that were used in further procedures.

For the creation of an ensemble model, only models whose predictive power is widely considered to be good were selected [73], i.e., models with ROC > 0.9 and TSS > 0.8. Based on several ensemble modeling strategies, the predictor variable importance was calculated and the species response curves were generated.

Projection of created ensemble model was done using current and future climatic conditions that rely on the different climatic models and emission scenarios.

The median of probabilities ensemble model strategy was selected, due to its more reliable predictive performance compared to others. Projected ensemble model rasters, whose outputs were binary transformed, were used for the calculation of species range change because the median is less sensitive to outliers than the mean.

Please, see Supplementary Material for more details.

## 5. Conclusions

Species distribution modeling is a useful tool for predicting areas to search for species that were previously unknown. In addition, the occurrence of B. viridis in Serbia is an indication that the survival of the species in the study area is unlikely without careful habitat conservation and management plans and programs. The occurrence of B. viridis is also highly determined by canopy cover and the amount of decomposing wood in suitable habitat forest types [75], suggesting that this species is an indicator of the functionality of certain forest ecosystem types and of good applied silvicultural methods and natural forest management. Declining population trends and prediction of habitat loss are the main reasons why the conservation status of this species in Serbia is still classified as Critically Endangered (CR), even though the number of recent detections in Serbia has increased.

## Data Availability

Not applicable.

## Supplementary Materials

The following supporting information can be downloaded at: https://www.mdpi.com/article/10.3390/plants12030557/s1, Occurrence_records—the occurrence records for the investigated species—*Buxbaumia viridis* (Moug. ex Lam. & DC.) Brid. ex Moug. & Nestl. On the territory of the Republic of Serbia; Current_climate—19 bioclimatic variables of current climatic conditions (1973–2013) in 30 arc-seconds spatial resolution; Future_climate—19 bioclimatic variables of predicted future climatic conditions. Three global climate models (CNRM-CM6-1; EC-Earth3-Veg and MIROC6) in two SSP scenarios (SSP2-4.5 and SSP5-8.5) for two reference periods (2050s i.e., 2041–2060 and 2070s i.e., 2061–2080) for the territory of the Republic of Serbia in 30 arc-seconds spatial resolution
