# Supplementary material for: Possible Effects of Climate Change on the Occurrence and Distribution of the Rare Moss Buxbaumia viridis in Serbia (SE Europe)
Source: Plants (Basel). 2023 Jan 26;12(3):557. doi: 10.3390/plants12030557 (PMC9921671; doi:10.3390/plants12030557)
Supplement: Supplementary file 1 [file plants-12-00557-s001.zip › readme_appendix.pdf]

Pantovic et al. (2023) Data from: Climate change will drastically affect the occurrence and distribution of rare moss Buxbaumia viridis in Serbia. Plants

Datasets

Here are the datasets used to (1) generate a species distribution model (SDM) and identify suitable and potential sites for *Buxbaumia viridis* in Serbia, from which the data about occurrence are still missing, (2) to assess environmental variables that have the greatest impact on its distribution in the country and finally (3) to predict its range in nearby future under predicted climate change.

Authors

Jovana Pantović

Institute of Botany and Botanical Garden, Faculty of Biology, University of Belgrade, Takovska 43, 11000 Belgrade, Serbia

jpantovic@bio.bg.ac.rs

Đorđe P. Božović

Institute of Botany and Botanical Garden, Faculty of Biology, University of Belgrade, Takovska 43, 11000 Belgrade, Serbia

djordje.bozovic@bio.bg.ac.rs

Marko S. Sabovljević

Institute of Botany and Botanical Garden, Faculty of Biology, University of Belgrade, Takovska 43, 11000 Belgrade, Serbia

Department of Botany, Institute of Biology and Ecology, Faculty of Science, Pavol Jozef Šafárik University in Košice, Mánesova 23, 040 01 Košice, Slovakia

marko@bio.bg.ac.rs

File folder contents

Occurrence_records – the occurrence records for the investigated species – *Buxbaumia viridis* (Moug. ex Lam. & DC.) Brid. ex Moug. & Nestl. On the territory of the Republic of Serbia

Current_climate – 19 bioclimatic variables of current climatic conditions (1973 – 2013) in 30 arc-seconds spatial resolution

Future_climate – 19 bioclimatic variables of predicted future climatic conditions. Three global climate models (CNRM-CM6-1; EC-Earth3-Veg and MIROC6) in two SSP scenarios (SSP2-4.5 and SSP5-8.5) for two reference periods (2050s *i.e.* 2041 – 2060 and 2070s *i.e.* 2061 - 2080) for the territory of the Republic of Serbia in 30 arc-seconds spatial resolution

File descriptions

Folder "Occurrence_records" includes a file " Buxbaumia_viridis_occurence_records.shp" an ESRI

shapefile with the occurrence records data for the investigated species with “lon” and “lat” columns (longitude and latitude, respectively)
BIO1 — Mean annual temperature in each grid cell (°C)
BIO2 — Mean diurnal range (mean of maximal temperature - minimal temperature) in each grid cell (°C)
BIO3 — Isothermality (Bio2/Bio7) (* 100) in each grid cell
BIO4 — Temperature seasonality (standard deviation *100) in each grid cell (°C)
BIO5 — Maximal temperature of the warmest month in each grid cell (°C)
BIO6 — Minimal temperature of coldest month in each grid cell (°C)
BIO7 — Temperature annual range (Bio5-Bio6) in each grid cell (°C)
BIO8 — Mean temperature of the wettest quarter in each grid cell (°C)
BIO9 — Mean temperature of the driest quarter in each grid cell (°C)
BIO10 — Mean temperature of the warmest quarter in each grid cell (°C)
BIO11 — Mean temperature of the coldest quarter in each grid cell (°C)
BIO12 — Total annual precipitation in each grid cell (mm)
BIO13 — Precipitation of the wettest month in each grid cell (mm)
BIO14 — Precipitation of the driest month in each grid cell (mm)
BIO15 — Precipitation seasonality (coefficient of variation) in each grid cell
BIO16 — Precipitation of the wettest quarter in each grid cell (mm)
BIO17 — Precipitation of the driest quarter in each grid cell (mm)
BIO18 — Precipitation of the warmest quarter in each grid cell (mm)
BIO19 — Precipitation of the coldest quarter in each grid cell (mm)
